# Supplementary material for: Crossing Bacterial Genomic Features and Methylation Patterns with MeStudio: An Epigenomic Analysis Tool
Source: Int J Mol Sci. 2022 Dec 21;24(1):159. doi: 10.3390/ijms24010159 (PMC9820200; doi:10.3390/ijms24010159)
Supplement: Supplementary file 1 [file ijms-24-00159-s001.zip › ijms-1997287-supplementary.pdf]

Table S1. Number of methylated sites detected in *S. meliloti* strains BE31LL, BO21CC, FSMA-MA and 1021. CDS, coding sequence; nCDS, coding sequence reverse strand; tIG, intergenic sequence between two genes in opposite directions; US, upstream sequence to a coding sequence. N.d., not detected.

	FSM-MA				2011				BE31LL				BO21CC			
**Motif**	CDS	nCDS	tIG	US	CDS	nCDS	tIG	US	CDS	nCDS	tIG	US	CDS	nCDS	tIG	US
ACGGAG	0.05	0.053	0.007	0.066	0.064	0.055	0.008	0.069	0.047	0.039	0.006	0.048	0.057	0.052	0.006	0.062
BNNCGATCGV	0.368	0.397	0.016	0.364	0.386	0.45	0.015	0.408	0.281	0.324	0.014	0.312	0.3	0.341	0.018	0.338
BYCGATCG	0.08	0.119	0.008	0.125	0.091	0.11	0.005	0.114	0.028	0.053	0.003	0.055	0.058	0.084	0.004	0.085
CCCGGG	0.026	0.035	0.003	0.042	0.033	0.043	0.003	0.046	0.012	0.02	0.001	0.024	0.025	0.04	0.003	0.047
CGATCGV	0.405	0.402	0.019	0.372	0.409	0.426	0.016	0.387	0.293	0.323	0.015	0.31	0.334	0.338	0.02	0.341
CTCGAG	0.143	0.137	0.008	0.144	0.127	0.17	0.014	0.176	0.087	0.066	0.003	0.08	0.087	0.092	0.012	0.113
CTYCCAG	0.014	0.028	0.002	0.031	0.02	0.051	0.004	0.058	N.d.	N.d.	N.d.	N.d.	N.d.	N.d.	N.d.	N.d.
DCTGCAGGS	0.013	0.015	0.001	0.017	N.d.	N.d.	N.d.	N.d.	0.012	0.013	0.002	0.015	0.219	0.184	0.019	0.203
GANTC	4.193	4.193	1.59	2.719	4.196	4.196	1.575	2.731	4.516	4.516	1.749	2.968	4.368	4.37	1.658	2.827
GCCAGG	0.022	0.05	0.003	0.052	0.084	0.111	0.004	0.113	2.043	2.41	0.264	1.517	0.032	0.055	0.006	0.059
GCCGGCH	0.36	0.292	0.028	0.294	0.436	0.412	0.032	0.384	0.286	0.237	0.029	0.258	0.299	0.297	0.03	0.303
GCCGGCYD	0.151	0.143	0.01	0.151	0.201	0.189	0.017	0.197	N.d.	N.d.	N.d.	N.d.	0.123	0.121	0.015	0.134
GCRDB	3.312	3.234	0.476	1.78	3.714	3.604	0.588	1.929	3.816	3.797	0.591	3.542	3.754	3.644	0.394	3.783
GNCGATCGVC	0.097	0.09	0.004	0.09	0.113	0.111	0.002	0.108	0.051	0.066	0.004	0.071	0.079	0.082	0.004	0.081
RAGCWGCTY	0.012	0.016	0.003	0.021	N.d.	N.d.	N.d.	N.d.	N.d.	N.d.	N.d.	N.d.	0.005	0.015	0.001	0.017
RCCAGCC	0.039	0.064	0.002	0.068	0.061	0.07	0.002	0.07	N.d.	N.d.	N.d.	N.d.	0.04	0.052	0.003	0.061
RCGATCGGC	0.066	0.025	0.002	0.027	0.059	0.03	0.003	0.036	0.052	0.022	0.001	0.024	0.055	0.029	0.003	0.033
RCTGCAGGS	0.013	0.014	0.001	0.016	N.d.	N.d.	N.d.	N.d.	0.011	0.012	0.002	0.014	0.16	0.106	0.01	0.118
RGATCY	0.061	0.062	0.003	0.063	0.084	0.092	0.009	0.106	0.051	0.026	0.003	0.032	0.082	0.059	0.004	0.063
SCTCGAG	0.112	0.114	0.007	0.117	0.107	0.163	0.011	0.167	0.07	0.051	0.002	0.061	0.071	0.075	0.012	0.092
TCGWCGA	0.291	0.224	0.008	0.222	0.197	0.132	0.009	0.143	0.142	0.097	0.008	0.109	0.187	0.129	0.009	0.136
VGCCGGCCC	0.011	0.016	0.002	0.019	0.02	0.025	0.003	0.03	0.009	0.01	0.001	0.012	0.007	0.019	0.001	0.022
VNCGATCGV	0.396	0.388	0.015	0.36	0.419	0.431	0.016	0.392	0.295	0.316	0.015	0.307	0.334	0.332	0.017	0.331

YCGATCGD	0.094	0.127	0.009	0.127	0.08	0.1	0.003	0.095	0.05	0.078	0.005	0.079	0.076	0.093	0.005	0.094
YCGGCCGRV	0.123	0.135	0.016	0.153	0.158	0.158	0.012	0.171	0.115	0.11	0.008	0.118	0.126	0.126	0.009	0.136
YCTGCAG	0.041	0.045	0.001	0.048	0.051	0.054	0.005	0.061	0.029	0.03	0.004	0.036	0.742	0.805	0.07	0.683
